# Cognitive Interventions: Symptomatic or Disease-Modifying Treatments in the Brain?

**DOI:** 10.14283/jarlife.2024.8

**Published:** 2024-05-22

**Authors:** F. Bellelli

**Affiliations:** 1. Fellowship in Geriatric and Gerontology, University of Milan, Milan, Italy; 2. Gérontopôle de Toulouse, Institut du Vieillissement, Centre Hospitalo-Universitaire de Toulouse, Toulouse, France.

**Keywords:** Cognitive stimulation, cognitive rehabilitation, cognitive training, Alzheimer disease, disease-modifying treatments

## Abstract

Recent findings suggest that brain-stimulating activities may have beneficial effects on both Mild Cognitive Impairment (MCI) and Alzheimer’s Disease (AD). However, whether cognitive interventions merely enhance cognitive reserve or truly attenuate, or even reverse, the disease’s pathophysiology is still controversial. The aim of the present article is to discuss the potential for brain-stimulating activities, including cognitive stimulation (CS), cognitive rehabilitation (CR), and cognitive training (CT), to be symptomatic or disease-modifying interventions in the context of cognitive decline. While emerging evidence indicates that CT can enhance synaptic plasticity, suggesting a potential role in augmenting cognitive reserve, its impact on AD pathology remains uncertain. Small-scale studies suggest that CT and CS may slow down neurodegeneration in MCI patients and that multidomain interventions combining physical activity with CT may benefit Aβ pathology. However, the considerable heterogeneity across studies limits the comparability of findings. It underscores the necessity for a more standardized approach to cognitive interventions in future guidelines for preventing and managing cognitive decline.

**T**he World Health Organization (WHO) and the National Institute for Health and Care Excellence (NICE) recommend social interactions and other brain-stimulating activities as non-pharmacological treatments for dementia (1, 2). However, the extent to which such interventions merely alleviate signs and symptoms of cognitive decline, reduce the pathophysiological burden of Alzheimer’s disease (AD – most prevalent type of dementia), benefit both aspects or have no discernible effect remains unclear and deserve further debate. The present article aims to discuss the potential for brain-stimulating activities to be symptomatic or disease-modifying interventions in the context of cognitive decline during aging.

## Brain stimulating activities

Among various brain-stimulating interventions, most recommendations endorse cognitive stimulation (CS) (1, 3), cognitive rehabilitation (CR)(1), or cognitive training (CT) (4). CS consists of various activities and discussions aimed at improving social and cognitive functioning (1). Similarly, CR works to achieve goals relevant to the person living with dementia and his family, trying to enhance and maintain functioning in everyday life (1). On the other hand, CT is a more specific approach that works on a set of standardized tasks designed to reflect singular cognitive functions (i.e., episodic memory) (1) and is, therefore, particularly suitable for individuals with Mild Cognitive Impairment (MCI) (5).

Multiple systematic reviews have suggested in the last two decades that individuals with MCI may experience slightly to moderate improvements following cognitive interventions. Still, the heterogeneity of the studies made it challenging to distinguish which method had the strongest impact (6, 7). Recently, a systematic review and meta-analysis extended the evaluation of cognitive interventions to individuals with AD dementia. Analyzing 25 studies involving 2012 participants, the review concluded that while further research on CR and CS is warranted, there is some indication of temporary benefits on global cognitive function following CT (8).

However, the mechanisms by which these interventions may improve cognitive functions are still debated. Indeed, according to the cognitive reserve hypothesis, individuals with a greater cognitive reserve can withstand a higher AD pathological burden before developing dementia by employing mental processing approaches or compensatory brain networks (synaptic plasticity) (9). Given that, do cognitive interventions enhance cognitive reserve, or do they genuinely attenuate or reverse the disease’s pathophysiology?

## Tackling cognitive decline symptoms through improved cognitive reserve

Recent findings suggest that cognitive interventions might have a beneficial impact on synaptic plasticity and, consequently, on cognitive reserve. Indeed, several studies have demonstrated that CT can enhance regional activity in functional Magnetic Resonance Imaging (fMRI) scans of patients with MCI following an intervention ranging from 2 weeks to 12 months (10–13). In particular, Hampstead et al. proposed that the most robust training-specific increases occur within areas of the default network that are abnormal in MCI and AD (medial frontal and parietal cortices and around the temporoparietal junction) (12). Accordingly, two studies found that compared to standard care, 8-week CR and 7-week CS programs could improve fMRI even in individuals with mild dementia (14, 15). Interestingly, Bentham et al. observed that individuals with high vascular burden had a lower functional connectivity response to CS than those with low burden, suggesting that vascular pathology could limit the potential for a neuroplastic response to cognitive interventions (16).

## Brain stimulation and the biomarkers of AD

Regarding the core biomarkers of AD (Aβ and Tau), evidence on cognitive interventions remains scarce and inconclusive. A small study employed a questionnaire to retrospectively evaluate the engagement in cognitively stimulating activities (e.g., reading, writing, playing games) across the lifespan of 65 healthy older adults and 10 AD patients. The study observed greater participation in cognitively stimulating activities, particularly during early and middle adulthood, was associated with decreased amyloid PET burden compared to individuals with limited involvement in such activities (17). Accordingly, secondary analyses of a subset of the Multidomain Alzheimer Preventive Trial (MAPT), an extensive study on community-dwelling older adults at risk of cognitive decline, suggested that a three-year multidomain intervention, including CT and physical activity (PA) advice, was associated with lower Aβ burden on amyloid PET compared to controls (18). In contrast, no significant difference was observed in plasma phosphorylated-tau levels (19). However, a small study on community-dwelling older adults (n=27) suggested that PA rather than CT may primarily drive the effects on Aβ levels. Indeed, the study reported reduced plasma Aβ levels after a 12-week program in both single-task (PA) and dual-task training groups (PA + CT), with no between-group difference (20).

On the other hand, a similar study on individuals with AD (n=34) observed that an 8-week dual-task training significantly lowered plasma Aβ levels. In contrast, no significant difference was reported for the group doing only PA, suggesting that CT might influence AD pathophysiology (21). Notably, a study on individuals with MCI assessing the effects of a 9-month cognitive intervention alone (mindful awareness practice; MAP) on Aβ-42 levels (salivary sample) found no significant differences between the treatment arm (MAP) and the active control group (22). Even in preclinical AD models, evidence remains unclear, with some studies suggesting decreased (23-25), no change (26, 27), or increased (28) Aβ load in transgenic mice exposed to cognitive stimulation (i.e., enriched environment or spatial training).

## Brain stimulation and the biomarkers of neurodegeneration

According to the Revised Criteria for Diagnosis and Staging of Alzheimer’s Disease, anatomic MRI and Fluorodeoxyglucose Positron Emission Tomography (FDG-PET) are biomarkers of non-specific degeneration in AD pathophysiology (29). However, cognitive interventions’ effects on structural and metabolic changes in the brain are still unclear. A retrospective study involving 329 cognitively unimpaired middle-aged adults revealed that individuals engaging in cognitively stimulating activities, such as playing card games, exhibited larger gray matter volumes in brain regions susceptible to AD pathology (30). In contrast, secondary analysis of a subset (n= 244) of the Finnish Geriatric Intervention Study to Prevent Cognitive Impairment and Disability (FINGER), a large-scale trial involving older adults at risk for dementia, found no changes in regional brain volumes or cortical thickness after two years of a multidomain intervention comprising PA, CT, diet, and vascular risk monitoring (31). The study suggests that multidomain intervention has no beneficial effect on neurodegeneration among cognitively unimpaired individuals at risk of AD. However, the design of the FINGER study did not permit the evaluation of the impact of interventions in individuals already experiencing some level of cognitive impairment. Three small-scale studies suggested that cognitive interventions may benefit MRI changes in individuals with MCI. Specifically, a small study allocated individuals with mild dementia to either a 7-week CS program (n=16) or standard care (n=13). The study observed that individuals in the treatment group maintained their total brain volume, as assessed by MRI, while those in the standard care group experienced a decrease (15). Accordingly, another study suggested that a 6-month multi-intervention program based on aerobic exercises and CS could decelerate atrophy in AD-related brain regions among MCI patients (32). Furthermore, the last study observed that undergoing 24 sessions of computerized CT led to focal increases in cortical thickness among individuals with MCI (33), even hinting at a potential for reversibility of neurodegeneration.

Regarding brain metabolism, some studies showed a trend toward regional metabolic changes following cognitive interventions among cognitively unimpaired individuals at risk for dementia. Secondary analyses of a subset population (n=67) of the MAPT study suggested that combined treatment with omega-3 supplementation and multidomain intervention (CT and physical activity; PA) did not significantly increase overall brain metabolism after six or twelve months of treatment. However, exploratory analyses employing voxel-wise approaches suggested that multidomain intervention could enhance metabolism in specific brain regions, including the right hippocampus, right posterior cingulate, left posterior para-hippocampal gyrus, and right insular cortex (34). Likewise, a small study on community-dwelling older adults (n=45) showed that a 16-week computerized CT had a trend toward a metabolic increase in the right inferior frontal gyrus without reaching the statistical significance (35). However, evidence in individuals with cognitive decline is limited and contradicting. A small study suggested that a six-month cognitive intervention might decelerate the widespread cortical metabolic decline in AD and MCI patients, with a more pronounced effect observed in the latter (36). On the contrary, another study observed that a 12-week home-based CT program did not yield significant improvements in brain metabolism in individuals with MCI (37).

## Conclusions

**Figure 1:**
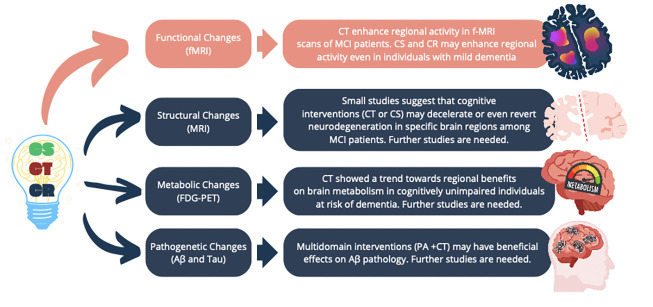
Effects of cognitive interventions on the brain

Legend: CS, Cognitive Stimulation; CT, Cognitive Training; CR, Cognitive Rehabilitation; fMRI, functional Magnetic Resonance Imaging; FDG-PET, Fluorodeoxyglucose Positron Emission Tomography; MCI, Mild Cognitive Impairment; PA, Physical Activity

In conclusion, evidence indicates a rise in synaptic plasticity following CT, suggesting that the beneficial effects of cognitive interventions may be partially attributed to the enhancement of cognitive reserve. However, whether cognitive interventions can attenuate or reverse AD pathophysiology (amyloid beta, tau, and neurodegeneration) remains subject to debate, and further studies are required (Figure 1). The need for robust evidence in this field could be attributed to various factors, including small study sample sizes, the substantial methodological heterogeneity across trials (rendering comparability across studies a problematic exercise), and combined interventions. Indeed, most studies evaluated the combined effects of physical activity and CT, making it challenging to discern the effects of one intervention. Additionally, the high cost of neuroimaging and limited accessibility to AD core biomarkers in cerebrospinal fluid (CSF) have posed significant constraints on large-scale population studies in previous years. However, the recent validation of more cost-effective and less invasive plasma-based biomarkers (29) may offer a valuable opportunity further to investigate cognitive interventions’ effects on AD pathogenesis.

Moreover, considering that cognitive interventions may enhance cognitive reserve, it is reasonable to expect different results based on participants’ baseline cognitive reserve. Therefore, further studies must consider at least the subjects’ educational level. Lastly, the primary reason for more evidence in the field might be the substantial heterogeneity of the studies. Notably, the duration (ranging from a few days to 3 years) and the methodologies employed in cognitive interventions were quite different between various trials. Indeed, although most guidelines endorse non-pharmacological approaches as first-line treatment for dementia (4) and MCI (38), official recommendations regarding the specifics of these interventions have often been lacking in previous years. Recently, the WHO ICOPE guidelines recommended cognitive stimulation for older adults with cognitive impairment, suggesting a standard group approach involving up to 14 themed sessions lasting approximately 45 minutes each, held twice a week (3). Continuing this path, future guidelines on the prevention of cognitive decline, as well as on the treatment and management of dementia, should aim to standardize at least the duration and the modalities of cognitive interventions while preserving the person-centered care approach that is a cornerstone of geriatric medicine.
